# Editorial:

**DOI:** 10.1120/jacmp.v12i4.3768

**Published:** 2011-11-15

With the publication of this issue of the *Journal of Applied Clinical Medical Physics*, we conclude our twelfth volume. Looking ahead to Volume 13, we will find several changes occurring with the Journal. The first of these changes is in the sponsorship of this periodical. As many of our readers may be aware, beginning on January 1, 2012, the American College of Medical Physics (ACMP), the sponsor of this Journal, will cease to exist, and many of the activities of the ACMP will be taken over by the American Association of Physicists in Medicine (AAPM). Beginning in 2012, the AAPM will sponsor two journals, a scientific journal, *Medical Physics*, and a clinical journal, the *JACMP*. Instead of reporting to the ACMP Board of Chancellors, the Journal's editorial board will report to the AAPM Board of Directors. However, you, as readers, will see no change in the nature of the manuscripts published in the *JACMP*, nor any change in the editorial policy, editorial procedures, or mission of the Journal.

A change that you will see, however, is that, beginning with Volume 13, we will be increasing our publication frequency to six issues per year, rather than four. Increasing our frequency of publication is a move that we have been considering for several years, and is a consequence of the increase in number of manuscripts submitted, and subsequently accepted, for publication. We believe the increase in publication frequency will have several advantages. First of all, the turnaround for accepted manuscripts will be faster. In order to meet our publisher's requirements, the deadline date for acceptance of a manuscript for publication in Volume 13 Number 1, scheduled for publication around February 15, is October 15, 2011. Any manuscripts accepted after that date would be published in Volume 13 Number 2. With our quarterly publication schedule, the publication date would be around May 15; hence, with the increased frequency of publication, the publication would occur one month earlier. A second benefit goes to our advertisers, whose message now can reach our readers six times a year, rather than four times a year. We hope that the decrease in turnaround time, coupled with the increased frequency of the Journal, will prove to be advantageous to both our readers and our advertisers.

Finally, I would like to acknowledge the difficult and time‐consuming work of our Associate Editors and reviewers in maintaining the high quality of manuscripts published in the *JACMP*. Names of Associate Editors can be found below, and names of reviewers can be found in a Supplementary File.

Associate Editors (including Guest Associate Editors) for Volume 12 include the following: Nzhde Agazaryan, Salahuddin Ahmad, John Antolak, Peter Balter, Pat Cadman, Marco Carlone, Nathan Childress, Geoff Clarke, Laurence Court, Luca Cozzi, Larry DeWerd, Bill Erwin, Kent Gifford, Rebecca Howell, Jennifer Johnson, Tommy Knöös, Stephen Kry, Harish Malhotra, Osama Mawlawi, Charles Mayo, Moyed Miften, Mike Mills, Eduardo Moros, Firas Mourtada, Ben Nelms, Jennifer O'Daniel, Tinsu Pan, Niko Papanikolaou, Donald Peck, Matt Podgorsak, Joann Prisciandaro, Jim Rodgers, John Rong, Yi Rong, Isaac Rosen, Surendra Rustgi, Bill Salter, Mehrdad Sarfaraz, Jeff Shepard, Chengyu Shi, Alf Siochi, Eric Slessinger, Jason Stafford, Lu Wang, Chuck Willis, Kamil Yenice, Al Zacarias, and Ron Zhu.

Thank you to our Associate Editors and our reviewers, and especially to our authors, without whose contributions we would have no Journal.

George Starkschall, PhD

Editor‐in‐Chief

November 15, 2011

